# Subaxial Subluxation (SAS) and Cervical Deformity in Patients with Rheumatoid Arthritis in Relation to Selected Sagittal Balance Parameters

**DOI:** 10.3390/jcm14144954

**Published:** 2025-07-13

**Authors:** Robert Wróblewski, Małgorzata Mańczak, Robert Gasik

**Affiliations:** 1 Department of Neuroorthopedics and Neurology Clinic and Polyclinic, National Institute of Geriatrics, Rheumatology and Rehabilitation in Warsaw, 1 Spartanska Street, 02-637 Warsaw, Poland; 2 Department of Gerontology, Public Health and Didactics, National Institute of Geriatrics, Rheumatology and Rehabilitation in Warsaw, 1 Spartanska Street, 02-637 Warsaw, Poland

**Keywords:** rheumatoid arthritis, SAS, skull, sagittal balance

## Abstract

**Introduction:** Synovitis and damage to natural stabilizers of many axial and peripheral joints make patients with rheumatoid arthritis particularly susceptible to sagittal balance disorders of the axial skeleton. This may determine the high individual variability of cervical spine deformities as well as differences in the rate of development of disease symptoms in these patients, such as radiculopathy and myelopathy. **Methods:** In the scientific literature, in addition to systemic factors, more and more attention is paid to work on biomechanical factors in the development of cervical spine instability. One of the methods for assessing the influence of biomechanical factors, which can also be used in everyday practice, is the analysis of radiological parameters of sagittal balance. **Results:** Among the selected sagittal balance parameters studied, a statistical relationship between C4 and C5 distance and the OI parameter has been found, indicating a relationship to a parameter that remains constant throughout an individual’s life in the group of patients with disease duration over 20 years. **Conclusions:** The development of instability and deformity in the subaxial segment of the cervical spine in patients with rheumatoid arthritis may be the result of insufficiently understood components of biomechanical factors; hence, further research in this field is necessary.

## 1. Introduction

Rheumatoid arthritis (RA) is a systemic autoimmune disease in which the immune response is directed against the synovial membrane of the joints [1]. The immunological process leads to the destruction of the joint surfaces of bones, ligaments, and tendons that are in contact with the synovial membrane. This results in, apart from nagging pain and swelling during periods of disease exacerbation, characteristic progressive joint deformations of the upper and lower limbs, especially in the hands and feet, and accompanying muscle atrophy [2]. After the small joints of the hands and feet, the third most common area of inflammatory changes in patients with RA is the joints of the cervical spine [3,4,5]. A. Garrod, who first described destructive changes in the cervical spine in RA in 1890, found them in 178 (35%) of 500 patients studied. According to more recent scientific publications, more than 80% of patients with RA have radiological involvement of the cervical spine, some already within 2 years of the initial diagnosis of RA [6,7,8]. The changes observed in everyday clinical practice in imaging studies of the cervical spine include erosion of the vertebral endplates, erosion of the sinus processus, ankylosis, apophyseal joint erosion or blurring, disc narrowing without osteophytosis, odontolite erosion, platybasia, fractures, and osteoporosis [9].

The effect of these changes is the loss of the compactness of the cervical spine joints, leading to disorders of the basic functions of the cervical spine. This results in an impairment of the effective protective and support functions of the cervical spine, especially because a simultaneous maintenance of the mobility is not found in other sections [10,11]. As a result of the damage, there are alterations in joint kinematics, such as disturbances in the range of motion from complete immobilization of segments due to ankylosis, to excessive mobility, instability, and subluxation of joints, leading to the development of the deformation [5,12].

**Figure 1:**
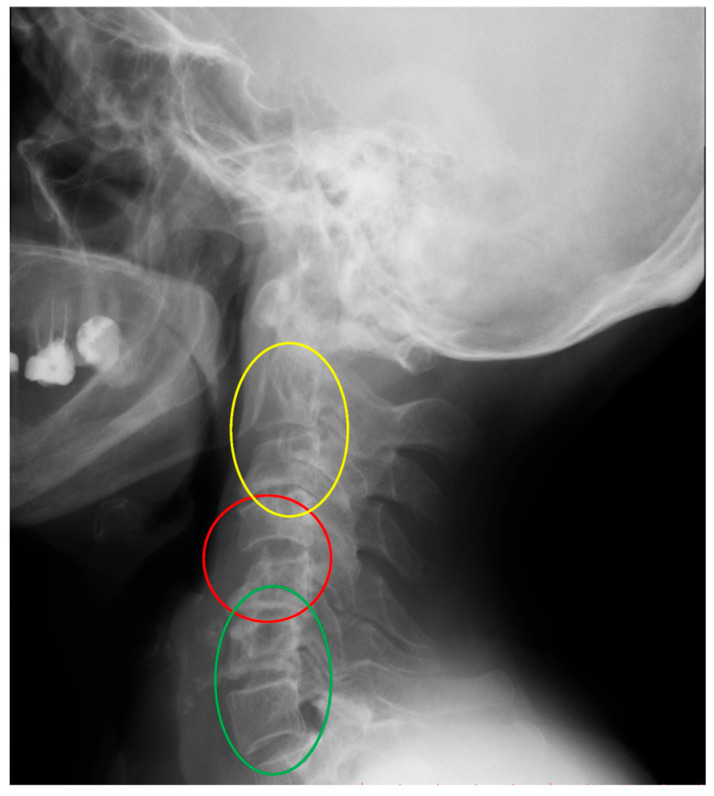
An X-ray of the cervical spine (lateral view) of a 75-year-old female patient with RA and SAS instability. C4–C5 instability (red). Cervical deformity with cervical lordosis below C4–C5 segment (green) and flatneck deformity above C4–C5 segment (yellow).

**Figure 2:**
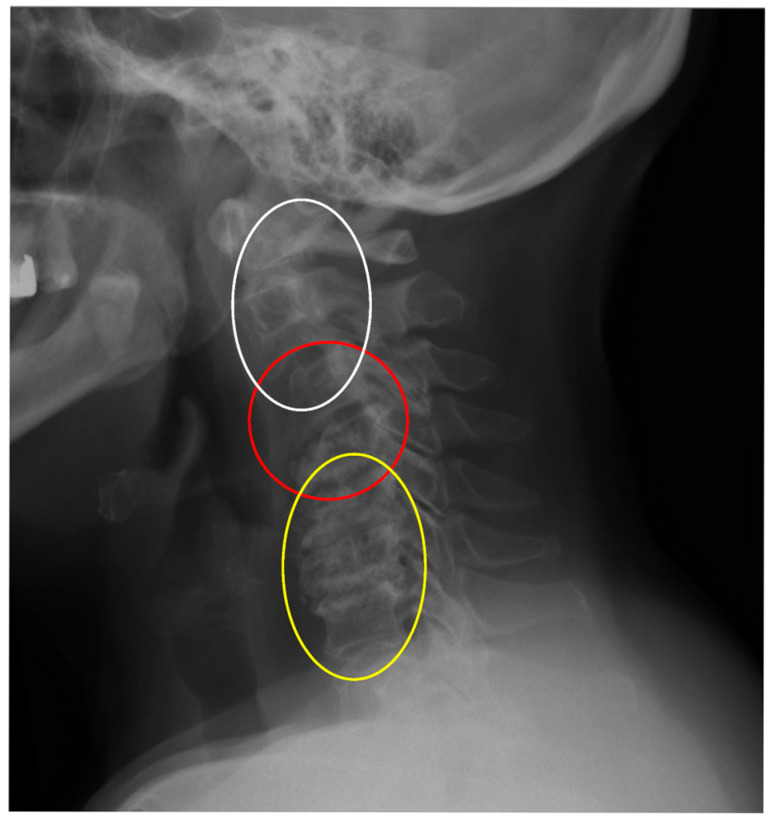
An X-ray of the cervical spine (lateral view) of a 67-year-old female patient with RA and SAS instability. C4–C5 instability (red). Cervical deformity with flat neck below the C4–C5 segment (yellow) and kyphotic deformity above the C4–C5 segment (white).

**Figure 3:**
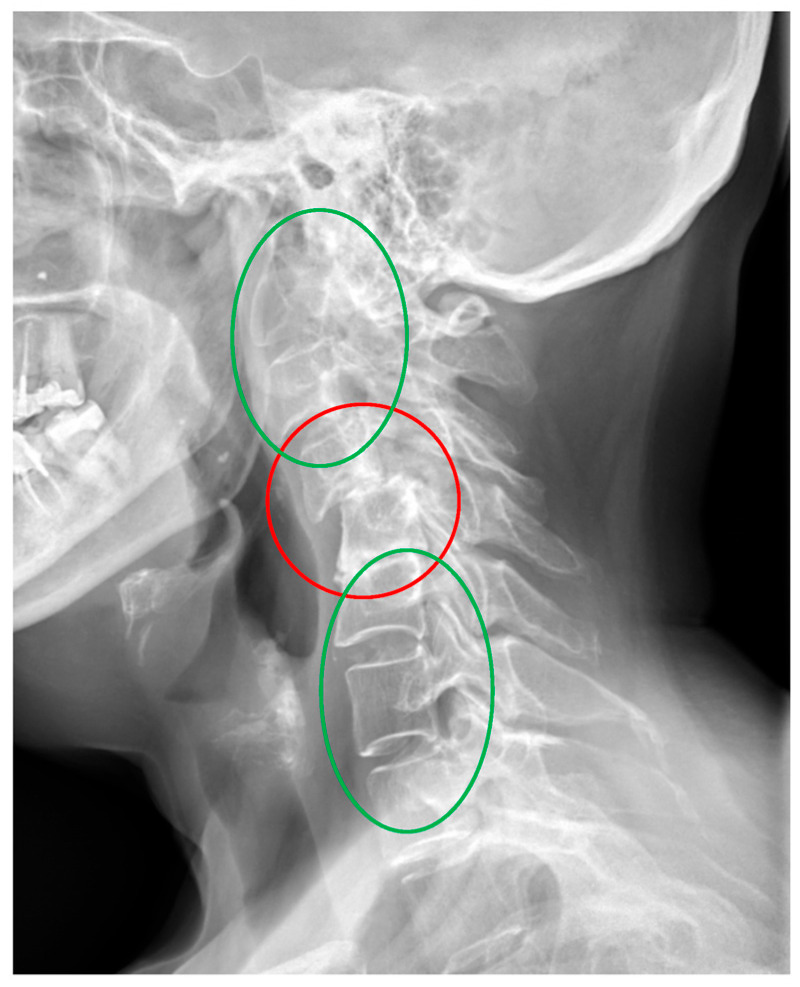
An X-ray of the cervical spine (lateral view) of a 54-year-old female patient with RA and SAS instability. C3–C4, C4–C5 instability (red). Cervical deformity with lordosis below C4–C5 segment (green) and lordosis above C4–C5 segment (green).

In clinical practice, it is typical to divide cervical dislocations into three types: instability AAS (atlanto-axial subluxation), SAS (subaxial subluxation), and CrS (cranial settling). (Table 1) [13,14]. This division is based on the anatomical differences in the affected areas. In this classification, SAS instability includes the vertebrae that constitute the five basic functional spinal units (FSU) of the spine, i.e., segments C2–3, C3–C4, C4–C5, C5–C6, and C6–C7. SAS instability is second only to AAS in terms of frequency of occurrence in patients with RA [13,14]. In daily practice, the greatest individual variety of deformities is observed in this area. They consist of abolition of lordosis, kyphoticization of the entire cervical segment or a part of it, deformities of a swan-neck nature, single- or multi-level instability, and anterior or posterior spondylolisthesis (Figure 1, Figure 2 and Figure 3).

Displacement of vertebral bodies relative to each other leads to canal and foramen stenoses, which can have a varied clinical picture, ranging from an asymptomatic course, through pain, vegetative disorders, dizziness, radiculopathy, myelopathy, to death as a result of compression of nerve structures or vessels [5,6,7,12]. We still do not know the answer to what determines the morphological variability of cervical segmental dislocations, which are responsible for various disease symptoms.

Scientific papers rarely address the issue of subaxial deformities in patients with RA. Scientific publications on this topic devoted to the surgical treatment of the cervical segment describe its inadequacies in long-term observations [15,16,17]. Cervical spine instability in patients with RA is a serious clinical problem. In addition to works focusing on improving pharmacological treatment procedures in RA, there are works on identifying factors associated with the development of cervical instability. Attention is paid to systemic factors such as disease activity, patient age, disease duration, gender, bone density, presence of the RF factor, and citrullinated antibodies [18,19,20,21,22,23].

The area of research that in recent years has contributed to expanding knowledge and understanding of the changes in selected spinal disorders and at the same time improving surgical planning is work on the sagittal balance of the spine [24,25]. The pioneering work on the sagittal balance was devoted to studies on the pelvis and lumbar spine [25,26]. A groundbreaking work, *Pelvic incidence: A fundamental pelvic parameter for three-dimensional regulation of spinal sagittal curves*, emphasizing the importance of the pelvis for sagittal balance, was published in 1998 by Legaye and Duval-Beaupère [27]. They drew attention to the basic value of the pelvic incidence (PI), the close relationship of this value with the sacral inclination, and the relationship of the sacral inclination with lumbar lordosis. In their concept, the pelvic morphology is individualized and personalizes the remaining spinal curvatures. Therefore, the pelvis was considered to be the regulator of the sagittal curvature of the spine [27]. These works inspired further research into the relationship between the lumbar spine and the thoracic spine, and then the thoracic spine and the cervical spine [28,29]. The collected results have led to the development of software such as Sugrimap, KEOPS, and Optispine for the sagittal analysis of the spine, enabling computer reconstruction and simulation of the reduction in surgical deformations, and consequently planning of a treatment strategy reducing the risk of complications [30]. Despite the advancement of knowledge, changes in the cervical spine in patients with RA still remain an area that eludes definition and poses a particularly serious therapeutic problem. The aim of this study is to search for biomechanical factors responsible for the development of cervical spine instability and deformation in patients with RA based on radiological parameters of sagittal balance.

## 2. Materials and Methods

This study included patients with diagnosed RA and confirmed cervical spine instability, who were consulted neuroorthopaedically for surgical treatment in the years 2019–2025 at the National Institute of Geriatrics, Rheumatology and Rehabilitation (NIGR & R). This study was retrospective, was approved by the hospital Ethical Committee (no. KBT-2/7/2019), and all patients gave their consent. It included a functional X-ray examination and a postural spine examination in the studied patients [31,32]. Radiological examinations were performed on the Carestream DRX Evolution—Health protocol. The diagnosis of RA was confirmed based on the EULAR/ACR guidelines from 2010 [33]. Cervical spine instability was differentiated according to the currently practiced anatomical division into AAS, SAS, and CrS. AAS was diagnosed for ADI (atlanto-dens interval—the distance between the anterior arch of C1 and the anterior surface of the odontoid) ≥ 3.5 mm, SAS (displacement of the endplates of the vertebral bodies relative to each other) for ≥2.5 mm or more, and CrS instability after meeting the criteria of Ranawat, Retlund-Johnell, and Clark [14,34,35,36,37,38,39]. In the scientific literature, the limit values for SAS are within the range of 2.0–3.5 mm. Due to the occurrence of myelopathy at SAS values of 2.0 mm, in order to eliminate interference related to measurement error, in our study, we assumed the limit of SAS instability to be 2.5 mm [14]. Patients with diagnosed instability additionally underwent a standing postural examination. Those were performed under constant conditions (by one radiologist) after adopting positions dedicated to postural examinations [40]. Exclusion criteria included the lack of a diagnosis of RA, patients with advanced disability that prevented radiological examination in a standing position without support, patients with a history of other cervical spondyloses, a tumor, congenital deformity, and other diseases that may lead to cervical spine instability, as well as infection, history of trauma, and previous surgical treatment.

**Figure 4:**
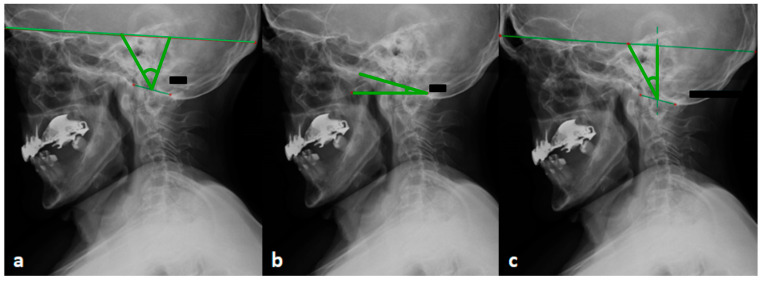
Skull parameters OI (**a**), OS (**b**), OT (**c**).

Radiological examinations were assessed in terms of sagittal balance parameters, which were divided into cranial, cervical, thoracic, and global parameters. Among the cranial parameters, the following were specified: OI, OS (in mod. W. Zhu), OT, and McGS [41,42]. McGS was used to determine CBVA [43] (Table 1, Figure 4).

**Table 1:** Radiological parameters within the skull.

Radiological Parameters Within the Skull	
Parameter	Definition
OI (occipital incidence)	An angle between the line connecting the center of the skull and the center of foramen magnum, and the line perpendicular to the foramen magnum
OS (occipital slope) (mod. in W. Zhu)	An angle between the line parallel to the foremen magnum and the horizontal line (according to the W. Zhu modification)
OT (occipital tilt)	An angle between the line directed through the center of the skull and the center of the foramen magnum and SVA
McGS (McGregor slope)	An angle between the line from the posterosuperior aspect of the hard palate and the caudal aspect of the opisthion and the horizontal
CBVA (chin-brow vertical angle)	An angle measured between the lines from the brow and the chin to the vertical

Selected radiological parameters in the cervical spine, thoracic, and global were COG-C7SVA (distance between vertical line of COG-center of gravity, to the vertical line SVA-sagital vertical axis to the center of C7), C2–C7SVA (distance from the posterosuperior corner of C7 to a vertical line from the center of the C2 vertebra), C1–C7 Cobb angle (the angle between the axis of C1 and superior endplate of C7), C0–C2 Cobb angle (the angle between the McRae’s line and inferior endplate surface of the C2), C2–C7 Cobb angle (the angle between the line passing through the lower endplate of C2 and superior endplate of C7), T1S—the angle between the upper endplate of T1 and the horizontal, in some C7S in some patients (the angle is formed between a horizontal line and the superior endplate surface of the C7), ThK (the angle between the superior endplate of the T1 and inferior endplate surface of the T12), and C7SVA HD (the distance between C7 SVA to the posterior arch of the upper sacral endplate surface).

The obtained results were then subjected to statistical analysis. Statistical analyses were carried out using STATISTICA v.13.1 (Statsoft; Dell Inc., Tulsa, OK, USA, 2016). The compliance of the distributions of the studied quantitative variables with normal distribution was examined using Kolmogorov–Smirnov tests. The distributions deviated from normal. Continuous variables are presented as median (Me) and interquartile range (IQR). Spearman’s correlation coefficients were used to assess the existence of relationships between quantitative variables. The limit of statistical significance was *p* < 0.05.

## 3. Results

This study included 54 patients with diagnosed RA: 45 (83.3%) women and 9 (16.6%) men. The mean age of women was 65 years (32 to 89 years); the mean age of men was 62 years (31 to 81 years). The median duration of the disease was 20 (12–30) years. SAS instability was divided into segments C2–C3, C3–C4, C4–C5, C5–C6, C6–C7. C4–C5 instability was the most common (36.5%) (Table 2). Isolated C4–C5 instability occurred in 4 study subjects, while in 19 cases it occurred together with instabilities at other C2–C7 levels.

The radiological assessment of the cervical spine took into account the level and degree (in millimeters) of instability, as well as the values of selected sagittal balance parameters. Results of the correlation analysis of C4–C5 values expressed in millimeters with other variables in the database (statistically significant values marked in red), in two groups: those suffering from the disease for over 20 years and up to 20 years (Table 3).

**Figure 5:**
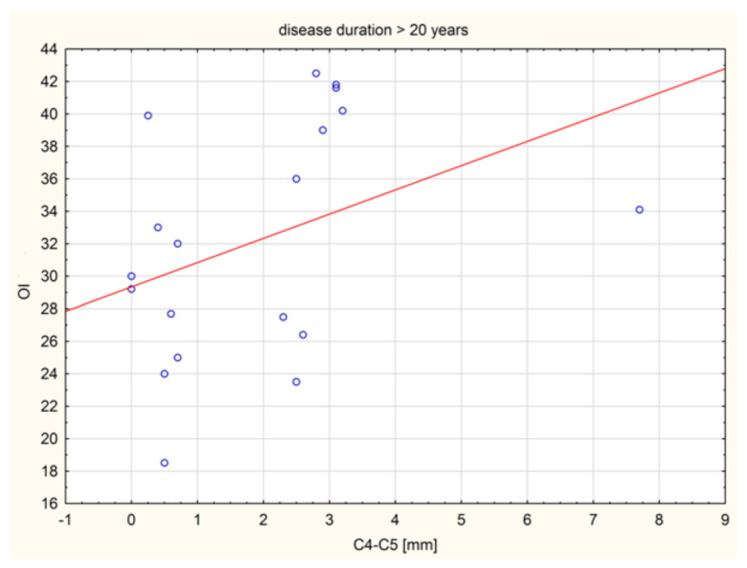
Scatter plots for C4–C5 (mm) and OI (deg.) for disease duration > 20 years.

A statistically significant positive relationship with moderate strength was shown between C4 and C5 (mm) distance in the group and the OI value in the group of patients with disease duration over 20 years. As the value of one variable increased, the values of the other variable increased (Figure 5).

## 4. Discussion

Studies on the sagittal balance of the cervical spine in healthy subjects and symptomatic patients have allowed for the introduction of sagittal balance parameters whose impact on the quality of human life has been well documented [10,28,29]. These include parameters such as C7SVA, C2–C7SVA, CBVA, and T1S–CL. They graphically reflect the biomechanical relationship between the spatial structure of the bone-ligamentous structures of the axial skeleton of the cervical spine and its neuromuscular regulation, with the development of pathology. The analysis of sagittal balance parameters has allowed for the discovery of mutual dependencies between adjacent sections of the pelvic, lumbar, thoracic and cervical spine as well as contributed to the creation of a classification of cervical spine deformities such as the Ames classification, and then, as in the work of Kim et al., led to attempts to standardize the stages of surgical procedure [44,45,46,47]. Synovitis leading to damage to the ligaments and joint capsules, destruction of the joint surfaces, the natural passive stabilizers of many axial and peripheral joints, makes patients with RA particularly susceptible to balance disorders and the development of disease symptoms [1,2,5,9]. Hence, it seems natural to discuss even those elements of the axial kinematic chain that have not attracted much attention so far, elements that may interact in these patients and be related to disease development. The results obtained in the statistical analysis have prompted us to draw attention to two issues that may shed new light on the knowledge of cervical deformities in patients with RA.

The first issue that prompts discussion is the role of the anatomy of the skull in the development of instability. The confirmed importance of the mutual relationships between adjacent spine sections encourages us to pay more attention to the elements that limit the cervical spine, i.e., the skull and thoracic section. From a biomechanical point of view, both sections, i.e., the skull and the thorax, are treated as rigid bodies and have an influence on the cervical spine that is similar to the influence of the pelvis and thorax on the lumbar spine. In the case of the thoracic spine, the mutual dependence of the thoracic and cervical spine is well known [48]. Among works on this issue, Knott et al. found that the spatial position of the cervicothoracic junction region may play an important role in the balance of the cervical spine, and proposed the T1 slope (T1S) parameter, a parameter comparable to the role of the sacral slope (SS) parameter in the balance of sagittal lumbar lordosis (LL) and pelvis [49]. In 2012, Lee et al. proposed the parameters of the thoracic inlet angle (TIA) and confirmed that the parameters of the TIA and T1S affect the sagittal balance of the skull and cervical spine [50]. Ames et al. confirmed the relationship of the cervical spine parameters and the T1S angle with the quality of life of patients and the development of disease symptoms [51]. In turn, the influence of the skull on the cervical spine is not as intuitive and obvious as that of the thoracic spine, due to the great mobility of the neck and the structure of the cervicocranial junction devoid of bony stabilizers. The work of J. Dubousset introduced the concept of the “cephalic vertebra”, defined as one of the elements of the kinetic chain, next to the “pelvic vertebra” [24]. While the role of the skull as a cephalic vertebra is not emphasized in scientific works as much as that of the pelvis, the skull, like the pelvis, is characterized by a great diversity of structure. With its contents, it can weigh, on average, from 4.5 to 5 kg, and according to the morphometric classification of Lebzelter and Sailer, the differences in the length measured from the glabella point to the occiput, as well as the width, can reach even more than 30 mm [52,53]. The skull as a solid is subject to the laws of kinetics. It is also the location of the organ of vision, the sense of balance, and proprioceptors, i.e., key elements of the regulatory mechanisms influencing horizontal vision and the play of postural muscles, described by J. Dubousset as the “harmony” of the human body [24,54]. Among the works on the role of the skull in sagittal balance are the works of Kimm et al. who, in 2013, described the parameter OI—an individually constant parameter, compared with the individual parameter PI of the pelvis, and the variable parameters OT and OS the algebraic sum of which equals OT + OS = OI (Table 1) [41,42]. In the work of W. Zhu et al., a close relationship between occipital parameters and cervical curvatures was found [41]. It was shown that the value of the OI angle is strongly correlated with the C0–C2 angle, the C2–C7 angle, and the C0–C7 angle. It was found that any change in one of these parameters causes a change in the others, except for the OI [41]. The results of the work of W. Zhu et al. showed that the sagittal position of the subaxial neck section was compensated by two factors, the thoracic factor and the occipital factor [41]. The current studies in the group of RA patients indicate a statistically positive relationship between the C4–C5 displacement and the OI value. Higher values of the OI angle correspond to greater distances between the perpendicular line drawn through the center of the foramen magnum and the center of the skull defined according to Kimm et al. [42]. This causes the moment arm of the force application to be longer and the energy expenditure to maintain it in a state of equilibrium to be greater. It should be emphasized that the parameters included in the classification of cervical spine deformities by Ames et al. do not directly reflect the significance of individual properties of the skull, which is the final element of the kinematic system of the axial skeleton [45]. Additionally, the C2–C7SVA, T1S–CL parameters reflect the spatial position of the cervical spine, without distinguishing between individual segments, which do not move smoothly and sometimes even in opposite directions [54]. In turn, the CBVA parameter, which is a strong determinant of the horizontal position of the skull, does not reflect its individual properties. Increasing the range of OI angle may require additional energy expenditure to maintain balance and horizontal vision, which may be important in patients with RA, in which, with the duration of the disease, progressive joint damage and global muscle weakness occur.

The second issue that the analysis of the results obtained in this study may draw attention to is the variability of the deformation of the subaxial region. The subaxial section consists of five similar anatomical functional units of the spine (C2–3, C3–4, C4–5, C5–6, C6–7). From a clinical point of view, the subaxial section is not homogeneous, the symptoms of radiculopathy at each level are different, but the main differences are that C2–C3 lesions mainly cause symptoms in the skull and occiput region, while pathological changes in segments C4 to C7 result in symptoms in various areas of the shoulder girdle and hands [5,13,55,56]. A factor indicating the heterogeneity of the subaxial section, emphasizing the role of neuromuscular regulation, is the characteristic pattern of neck movement. Flexion movement begins in segments C4–C7, i.e., is initiated in segment C6–C7 and continues in C5–C6 and C4–C5. The next stage in the sequence is C0–C2, and in a variable order, movement in the C2–C3 and C3–C4 segments. It can be seen that the flexion movement at the C4–C5 level loses its internal fluidity, moving and continuing in accordance with the pattern to the C0–C2 level [54,57]. In the work of Anderst et al. on the relationship between the position of the skull and the kinematics of the cervical spine, it was found that the change in the spatial position of the skull led to changes in the position of the cervical vertebrae, the greatest range of movement of which occurred precisely at the C4–C5 level [58]. This may provide an answer as to why, in patients with RA, the kinetic properties of the skull contribute to the occurrence of deformations in the joints at exactly this level. Permanent damage to the FSU at the C4–C5 level occurring with the duration of RA may be related to the disease itself, but also to an element that has not attracted much attention so far, such as the morphological properties of the skull, which may be reflected by the OI parameter. It seems that at this point, it is necessary to recall the results of previous studies published in articles on the relationship between pelvic parameters and cervical spine instability in patients with RA. The results showed a statistical relationship between C2–C3 instability and the pelvic parameter PT and a relationship between C1–C2 instability and OD-HA [59,60]. Comparison of the results obtained in our study with experimental studies is difficult because, in the available scientific works, there are few reports on this type of study of cervical spine deformities in RA. The published works focus on peripheral joints [61,62]. This is related to the difficulties in simulating complex, multi-site changes—examples of which are given in the introduction to this article—in laboratory conditions [3,5,9]. Finite Element Method (FEM) studies in degenerative changes, cervical spine injuries or in biomechanical analysis of disc arthroplasty do not reflect the complex specific properties of the osseous and ligamentous system of the spine in patients with RA [63,64]. An additional difficulty in reconstructing the complex deformities of the cervical spine, exemplified by the images presented at the beginning of this article (Figure 1, Figure 2 and Figure 3), is the suggestion that the changes observed in it are not the result of locally applied loads, as in experimental studies, but the resultant of complex compensatory mechanisms of the entire axial system and body striving to ensure the supportive and behavioral function of the skull, sometimes at the expense of losing the natural physiological curvatures of the cervical segment. This requires further in-depth studies. However, it can be assumed that the spatial position of the cervical spine, in addition to biomechanical factors reflected by parameters whose impact on quality of life has been confirmed, may also be a result of factors acting segmentally on the upper or lower parts of the subaxial region. In the future, such a complex approach may contribute to a full understanding of the mechanisms of development of cervical spine instability and deformation and facilitate the decision on treatment.

This study has several limitations. One limitation of this study is the small sample size, which affects the statistical power of the correlation coefficient, which was 60%. Therefore, the results should be considered as preliminary and require further validation in studies with larger samples. The second limitation is that the analysis does not account for potential confounders, such as medications, disease activity scores, or comorbidities, which may have influenced the results. Another limitation of this study is also its retrospective nature, which causes further limitations. What is more, this study did not include patients who, due to the advanced stage of the disease, could not assume a free vertical position without support. A further limitation is the lack of consensus on the definition of SAS instability diagnosis. Some authors diagnose SAS when the horizontal displacement of the vertebrae is more than 3.5 mm. In the search for OI values at which instability occurs, statistical significance was not obtained due to the small number of subjects, apart from the finding that patients with C4–C5 instability corresponded to higher OI values. This study is preliminary in nature and suggests potential directions for further research.

## 5. Conclusions

A statistically significant positive relationship was shown between C4–C5 (mm) distance and the OI value in the group of patients with disease duration exceeding 20 years. As the value of one variable increased, the values of the other variable increased. The relationship was statistically significant, positive, and of moderate strength.

## Figures and Tables

**Table 2:** Number of cases, SAS, and percentage of recognized instability.

Number of Cases SAS and Percentage of Recognized Instability.		
Type of Instability	Cases of Instability	Percentage
C2–C3	11	17.4%
C3–C4	17	27%
C4–C5	23	36.5%
C5–C6	10	15.9%
C6–C7	2	3.2%

**Table 3:** Spearman’s correlation coefficients.

Variable	Spearman’s Correlation Coefficients		
	Number of Cases *n* = 54	Patients with Duration of RA > 20 y *n* = 21	Patients with Duration of RA ≤ 20 y *n* = 24
COG–C7 SVA (mm)	−0.030	−0.051	−0.167
C2–C7 SVA (mm)	0.122	0.084	0.042
C7 SVA (mm)	−0.101	−0.085	−0.118
OI (deg.)	0.216	***0.477***	−0.197
OS(deg.) (mod. in W. Zhu)	−0.075	0.264	−0.302
OT (deg.)	0.201	0.313	0.021
McGS (deg.)	0.015	0.069	0.032
C1–C7 Cobb angle	0.091	0.243	0.022
C0–C2 Cobb angle	−0.192	−0.202	−0.254
C2–C7 Cobb angle	−0.061	−0.016	0.143
C7S (deg.)	0.053	0.122	0.092
T1S (deg.)	0.180	0.084	0.110
ThK Cobb angle	−0.142	0.148	−0.147

## Data Availability

The data are available from the corresponding author if required.
